# Evaluation of Goal Recognition Systems on Unreliable Data and Uninspectable Agents

**DOI:** 10.3389/frai.2021.734521

**Published:** 2022-02-02

**Authors:** Irina Rabkina, Pavan Kantharaju, Jason R. Wilson, Mark Roberts, Laura M. Hiatt

**Affiliations:** ^1^Computer Science Department, Occidental College, Los Angeles, CA, United States; ^2^Smart Information Flow Technologies, Minneapolis, MN, United States; ^3^Computer Science Department, Franklin & Marshall College, Lancaster, PA, United States; ^4^The U.S. Naval Research Laboratory, Washington, DC, United States

**Keywords:** goal recognition, reliability, inspectability, hierarchical task networks, combinatory categorial grammars, analogical reasoning

## Abstract

Goal or intent recognition, where one agent recognizes the goals or intentions of another, can be a powerful tool for effective teamwork and improving interaction between agents. Such reasoning can be challenging to perform, however, because observations of an agent can be unreliable and, often, an agent does not have access to the reasoning processes and mental models of the other agent. Despite this difficulty, recent work has made great strides in addressing these challenges. In particular, two Artificial Intelligence (AI)-based approaches to goal recognition have recently been shown to perform well: goal recognition as planning, which reduces a goal recognition problem to the problem of plan generation; and Combinatory Categorical Grammars (CCGs), which treat goal recognition as a parsing problem. Additionally, new advances in cognitive science with respect to Theory of Mind reasoning have yielded an approach to goal recognition that leverages analogy in its decision making. However, there is still much unknown about the potential and limitations of these approaches, especially with respect to one another. Here, we present an extension of the analogical approach to a novel algorithm, Refinement via Analogy for Goal Reasoning (RAGeR). We compare RAGeR to two state-of-the-art approaches which use planning and CCGs for goal recognition, respectively, along two different axes: *reliability* of observations and *inspectability* of the other agent's mental model. Overall, we show that no approach dominates across all cases and discuss the relative strengths and weaknesses of these approaches. Scientists interested in goal recognition problems can use this knowledge as a guide to select the correct starting point for their specific domains and tasks.

## 1. Introduction

Recognizing another agent's goals is key for many types of teamwork. Shoulder-to-shoulder teamwork requires knowing a teammate's goals so that one can either assist or, at a minimum, not detract from their progress (Geib et al., 2016). Virtual agents also benefit from knowing a teammate's goals; such an agent can, for example, facilitate a task by highlighting information relevant to a user's goals in a display.

In contrast to its utility, however, goal recognition is a very challenging problem to solve. There are many reasons behind this difficulty. Primary among them is that goal recognition agents, by nature of the problem, typically can only indirectly inspect another agent's intentions/goals/etc.; the goals of the observed agents need to be inferred via their actions and behaviors. This is made even more challenging by obstacles such as noisy sensor readings.

In this work, we assess how well goal recognition algorithms handle different levels of agent *inspectability* and different forms of data *reliability*. Specifically, we look at three levels of inspectability (low, medium, and high), which refer to the amount of unobservable information (i.e., information that is internal to an agent) that is available to the goal recognition algorithm. Lower inspectability means less internal information is available, such as only seeing outward behavior, while higher means more internal information, such as accessing the agent's thought processes. We also consider two forms of data reliability: missing actions and incorrect action parameters. Missing actions can occur if an agent either actively does not complete the action or completes it, but the action does not register due to a faulty sensor. Incorrect action parameters occur as result of perception failure or mistakes, such as confusing a cup with a jar.

Many different methods for goal recognition have been proposed in past work (Vered and Kaminka, 2017; Shvo et al., 2020), as well as methods for its sibling problems plan recognition (Ramírez and Geffner, 2009; Mirsky and Gal, 2016; Höller et al., 2018), activity recognition (Hussain et al., 2019), and theory of mind reasoning (Hiatt et al., 2011; Rabkina et al., 2017, 2020). In particular, three goal recognition methods have recently been shown to perform well: goal recognition as Hierarchical Task Network (HTN) planning (Höller et al., 2018), goal recognition as a language parsing via Combinatory Categorial Grammars (CCGs) (Steedman, 2001; Geib, 2009), and goal recognition via analogy (Rabkina et al., 2020). We focus on these methods.

The contributions of this work are two-fold. First, we introduce a novel goal recognition algorithm called Refinement via Analogy for Goal Reasoning (RAGeR). This method extends an existing approach for goal recognition via analogical reasoning, Analogical Theory of Mind (Rabkina et al., 2017, 2020) by allowing it to leverage pre-existing goal recognition models. Second, we conduct an evaluation of RAGeR and two state-of-the-art goal recognition algorithms, PANDA-Rec (Höller et al., 2018) and Elexir-MCTS (Kantharaju et al., 2019), on data with varying inspectability and reliability from the open-world computer game Minecraft, and from the disaster management domain Monroe. We find that each approach to goal recognition has their own strengths and weaknesses. Specifically, our results indicate that PANDA-Rec performs well-compared to RAGeR and Elexir-MCTS on data with high inspectability, while Elexir-MCTS performs better with data on medium to low inspectability. However, we see that unreliabilty resulting from incorrect action parameters and missing noise on data from Minecraft and Monroe decreases the performance of Elexir-MCTS, PANDA-Rec, and RAGeR, but RAGeR's performance decreases more slowly than that of Elexir-MCTS and PANDA-Rec. We hope these results inform the community about the conditions under which a particular approach works well, so as to help guide others in choosing a goal recognition algorithm.

## 2. Related Work

Goal Recognition is the process of inferring the top-level goal of a partial plan executed by an agent (Mirsky et al., 2021) and is of interest to a variety of AI-related research communities and topics, including cognitive science (Rabkina et al., 2017), gaming (Gold, 2010), human-robot teaming (Hiatt et al., 2017), and others. Related work falls along two axes: techniques for goal recognition, and assumptions placed on the information available to goal recognition. We will return to these axes in section 4.1 to describe how our work helps to better describe the relative strengths and weaknesses of the approaches in different types of situations.

### 2.1. Goal Recognition Techniques

While there are many types of approaches that can be used for goal recognition, we focus on four conceptually different approaches here: theory of mind-based approaches, plan-based goal recognition, goal recognition as planning, and learned goal recognition.

#### 2.1.1. Theory of Mind-Based Approaches

Work in theory of mind, which can include inferring another agent's intentions (i.e., goals), has yielded rich computational models that can model human judgments (Baker et al., 2011; Hiatt et al., 2011; Rabkina et al., 2017). Görür et al. (2017) is one approach that performs theory of mind-based intent recognition that incorporates a human's emotional states into its recognition, focusing on determining when a human may or may not want a robot's assistance with their task. Our work, in contrast, focuses on improving the accuracy of the recognition step itself.

#### 2.1.2. Case-Based Reasoning

Goal recognition can also be done via case based reasoning (CBR) as demonstrated by Cox and Kerkez (2006) or Fagan and Cunningham (2003). Such approaches use case libraries that store sets of actions or observations of an agent along with the goal that the agent was accomplishing while performing those actions. The case libraries can be learned incrementally over time (Kerkez and Cox, 2003), and so do not always explicitly model an agent's behavior. When trying to recognize a goal, these approaches retrieve a case from their library that best matches the current situation, and use the goal of that case as the recognized goal. This is similar in spirit to one of the approaches we discuss here, RAGeR; however, RAGeR is unique in that its retrieval mechanisms is based on cognitive analogy.

#### 2.1.3. Plan-Based Goal Recognition

Plan-based goal recognition approaches generally utilize a library of the expected behaviors of observed agents that is based on a model of its behavior. These libraries have been represented in a variety of ways, including context-free grammars (CFGs) (Vilain, 1990), probabilistic CFGs (Pynadath and Wellman, 2000), partially-ordered multiset CFGs (Geib et al., 2008; Geib and Goldman, 2009; Mirsky and Gal, 2016), directed acyclic graphs (Avrahami-Zilberbrand and Kaminka, 2005), plan graphs (Kautz, 1991), hierarchical task networks (HTNs) (Höller et al., 2018), and combinatory categorial grammars (CCGs) (Geib, 2009). The last two are among the most popular, which is why PANDA-Rec and Elexir-MCTS, two of the approaches we explicitly analyze in this paper, are based on them.

#### 2.1.4. Goal Recognition as Planning

Goal recognition as planning (e.g., Hong, 2001; Ramírez and Geffner, 2009; Ramirez and Geffner, 2010; E-Martin et al., 2015; Sohrabi et al., 2016; Vered and Kaminka, 2017; Pereira et al., 2020; Shvo and McIlraith, 2020; Shvo et al., 2020) do not use explicit plan libraries. These approaches use off-the-shelf classical planners to solve the goal recognition problem. Generally, when recognizing goals, these approaches generate plans for different possible goals and see which best match the observed behavior. The main advantage is that they then require only a model of the domain's actions instead of one that explicitly contains the expected behavior of observed agents. However, they are not always robust to differences between the generated plan and the executed plan.

#### 2.1.5. Learned Goal Recognition

Gold (2010) uses an Input-Output Hidden Markov Models (Bengio and Frasconi, 1994) to recognize player goals from low-level actions in a top-down action adventure game. Ha et al. (2011) uses a Markov Logic Network (Richardson and Domingos, 2006) to recognize goals in the educational game Crystal Island. Min et al. (2014) and Min et al. (2016) use deep learning techniques (i.e., stacked denoising autoencoders, Vincent et al., 2010; and Long Short-Term Memory, Hochreiter and Schmidhuber 1997) to also recognize goals in Crystal Island. Pereira et al. (2019) combine deep learning with planning techniques to recognize goals with continuous action spaces. Amado et al. (2018) also use deep learning in an unsupervised fashion to lessen the need for domain expertise in goal recognition approaches; Polyvyanyy et al. (2020) take a similar approach, but using process mining techniques. To learn these models, existing data of agents' behaviors is required to learn these models. In our approach, in contrast, we use domain knowledge to construct a model and so do not require this learning.

### 2.2. Characteristics of Goal Recognition Data

We consider here work related to what data is available for goal recognition. Specifically, we consider levels of inspectability of the other agent's mental model in the data and levels of reliability of the observations that comprise the data.

With respect to inspectability, most approaches evaluate on data that has a constant level of agent inspectability. Generally speaking, that is at the level of knowing the actions that an agent takes (vs. the full plan, or vs. only observing their behavior). We therefore focus this discussion on work related to the reliability of data.

As with inspectability, most prior work uses a single dataset with a particular set of characteristics (whether reliable or not) to evaluate competing goal recognition approaches. Sohrabi et al. (2016) provides one exception to this, and considers unreliable observations that can be missing or noisy (i.e., incorrect). They show that noisy observations can, for some approaches that perform goal recognition as planning, prove more challenging than missing observations; this can be mitigated, however, by adding penalties for missing or noisy observations into the “costs” that rank candidate plans. Borrajo and Veloso (2020) handle such noise by using plan-based distance measures between observed execution traces and candidate plan traces. We also consider these two types of reliability in our experiments.

Ramirez and Geffner (2011) look at how a partially-observable Markov decision process (POMDP) performing goal recognition can handle missing or noisy observations, in part because of its probabilistic representation of agent behavior. POMDPs can be fairly computationally expensive to compute, however, precluding our use of them here.

Another prior study that looked at inspectability compared a goal recognition via analogy approach, Analogical Theory of Mind (AToM) with an HTN-based goal recognition approach (Rabkina et al., 2020). It showed that while the HTN-based approach performed better under high inspectability, the HTN-based approach degraded quickly as inspectability lessened, while AToM maintained a fairly high accuracy throughout. We include the same HTN-based approach, PANDA-Rec, in this paper, as well as RAGeR, a goal recognition approach that is an extension of AToM.

A long line of work focuses on learning action models from partial or noisy traces. Wang (1995) created a system to learn the preconditions and effects of STRIPS planning operators from expert traces and demonstrated that having the system refine the learned knowledge was able to obtain results as good as expertly crafted operators. Amir and Chang (2008) develop a method for online, incremental learning of action models for partially observable deterministic domains. They demonstrate that the approach can learn exact action models from a partially visible subset of the traces from benchmark PDDL problems from the 1998 and 2002 International Planning Competition. Mourao et al. (2012), in turn, are able to learn STRIPS planning operators from noisy and incomplete observations by using classifiers, which are robust to noise and partial observability, as an intermediate step in the translation. Pasula et al. (2007), in contrast, look at learning symbolic models of domains with noisy, non-deterministic action effects. Plan rules are both relational and probabilistic, and are learned by selecting the model that maximizes the likelihood of the input action effects. Zhuo and Kambhampati (2013) consider how to learn action models where actions are not always correctly specified (i.e., “pickup” instead of “putdown”). Gregory and Lindsay (2016) developed an approach for the automated acquisition of models for numeric domains (such as tracking resource usage). Related approaches also can operate when their underling *model* may not be correct, and take steps to update it iteratively during execution (Chakraborti et al., 2019). While we assume that the models used by the three approaches we consider are pre-existing and correct, this prior work could be incorporated into the approaches discussed here to initially learn the domain models, or to improve their model and goal recognition over time.

## 3. Goal Recognition Approaches

We define the *goal recognition problem* as a 4-tuple (*D*, **o**, *s*_0_, *G*), where *D* is a recognition model which encodes the expected plans that an observed agent would pursue for any known goal, **o** = 〈*o*_1_, …, *o*_*i*_〉 is sequence of observed actions, *s*_0_ is the initial state in which **o** was executed, and *G* is a set of known goals. We note that action models (i.e., preconditions and effects) are optional for actions in *D*. Goal recognition can still be done even if the action models do not exist. The *solution to the goal recognition problem* is a goal *g*∈*G* that is being pursued in **o**. Goal recognition approaches differ in how they fulfill and represent *D*, the recognition model. Thus, using this definition, we next present the three state-of-the-art approaches to goal recognition that we compare in this article.

### 3.1. Goal Recognition via Hierarchical Task Network Planning

To perform goal recognition as planning, we use a Hierarchical Task Network (HTN)-based planning algorithm called Planning and Acting in a Network Decomposition Architecture (PANDA)^1^ (Bercher et al., 2014). Hierarchical Task Networks are a type of plan representation where higher-level goals or tasks decompose into component subgoals or subtasks in a tree-like structure; the “leaves” of the tree serve as primitive actions that can then be sequentially executed to achieve the top-level goal. The primitive actions in the HTNs used by PANDA are *typed* in that the parameters of each primitive action is associated with a type characterizing objects in a domain (e.g., the action **move** takes an object of type agent). PANDA-Rec is a goal recognition algorithm that uses PANDA in its reasoning. Fundamentally, PANDA-Rec fulfills *D* by, at run time, generating candidate plans that both accomplish known goals and match the agent's behavior. To do this, the search for plans that is typical for HTN planning is constrained such that any candidate plan found must begin with **o**. In other words, PANDA-Rec finds goals that match the agent's behavior by enforcing a prefix requirement when matching candidate plans to an agent's observed behavior, where **o** exactly matches either an entire candidate plan or the beginning of a candidate plan for any given goal in the planning domain. If there is only one such goal, that is returned as the recognized goal. Otherwise, PANDA internally breaks the tie (see Höller et al., 2018 if interested in this tie-breaking process).

Throughout this section, we will refer to a running example from the game of Minecraft, which is an open-world sandbox world where a character collects resources to build items that are used to progress the game further. We will focus our example on how an agent collects wheat to craft bread. Usually, the agent will need to plant wheat seeds, apply bonemeal to encourage fast growth, strike the mature wheat to harvest it, and then gather the wheat from the ground. However, the gathering step can sometimes happen automatically if the agent is near enough to the wheat after harvesting it. Once gathered, three wheat can then be used to construct bread. To accomplish the use of an item in inventory, the agent must select and use it. There are similar recipes for harvesting other food items in the game.

As an example, suppose PANDA-Rec observes that an agent in the open-world computer game Minecraft wants to obtain bread, and thus harvests and gathers two wheat [i.e., **o** is **harvest(wheat), gather(wheat), harvest(wheat), gather(wheat)**]. Next suppose we have an HTN that defines the plan for obtain bread as **harvest(wheat), gather(wheat), harvest(wheat), gather(wheat), harvest(wheat), gather(wheat)**. The observed actions **o** matches the prefix for the plan of obtain bread, and so PANDA-Rec will return that as the recognized goal. However, suppose that the agent harvests two wheat without gathering them, such as if the agent wanted to harvest multiple wheat before gathering them (i.e., **o** is **harvest, harvest**). This will not match a candidate plan (or plan prefix) for obtain bread and PANDA-Rec will fail to recognize the agent's goal.

We note that for each action in **o**, it is crucial that the types of the action's parameters match the types of its corresponding primitive action in the HTN; PANDA-Rec will fail to recognize a goal if there is a type mismatch. For example, if the primitive action **harvest** expects a parameter of wheat type [e.g., **harvest(wheat)**], but the action in **o** contains a parameter of type animal [**harvest(chicken)**], then PANDA-Rec will fail to recognize a goal as a chicken is not a type of wheat.

While we have described here the details of PANDA-Rec that are critical for our evaluation and discussion, for interested readers, the full details of this process are described by Höller et al. (2018).

### 3.2. Goal Recognition via Combinatory Categorial Grammars

Elexir-MCTS (Monte-Carlo Tree Search) solves goal recognition as a form of language parsing (Vilain, 1990), where the model *D* is fulfilled via a Combinatory Categorial Grammar (CCG) (Geib, 2009). The CCG is an expressive grammar formalism made up of a finite set of rules that combine the semantic and syntactic structure of plans. CCGs can naturally represent interleaved plans for similar or different goals, and can efficiently capture plans or actions that can be done in any order. One example of interleaved plans in the computer game Minecraft would be an agent doing a plan to obtain bread while simultaneously also doing a plan to obtain potato (such as by gathering wheat and a potato before actually making the bread). Elexir-MCTS has demonstrated strong performance and improvement in scaling goal recognition over comprehensive search (Kantharaju et al., 2019); this recent success led us to use Elexir-MCTS in our study.

During goal recognition, Elexir-MCTS leverages the rules of the CCG to recognize the goals behind observed actions. Importantly, the rules of the CCG are specific pairs of actions and plans, and encode parts of the plan that need to be seen prior to executing the action (i.e., the plan prefix) and parts of the plan that should be done after executing the action (i.e., the plan suffix). Using these prefix/suffix rules, Elexir-MCTS searches for a set of weighted hypotheses *E*, where each *e* ∈ *E* is a rule that may correspond to what is being executed in **o**. Intuitively, a rule is included in *E* if, at minimum, the plan prefix of the rule matches some *subsequence* of **o**; failure to match the prefix of a rule will result in the rule not being included in *E*. Any part of the plan suffix that is matched additionally increases the weight of the rule.

Elexir-MCTS then uses these weighted rules to make an informed decision about the goals being pursued by the agent. Specifically, Elexir-MCTS computes a belief value for each goal *g* ∈ *G* by adding the weights of each rule in *E* that achieves *g* (i.e., each rule that corresponds with a plan that achieves *g*), and the highest-belief goal is returned as the goal of **o** (see Geib, 2009 for information on how the belief values and weights for each hypothesis are computed).

While, on its surface, Elexir-MCTS may seem to enforce a strict prefix requirement as PANDA-Rec did above, its representation of *D* as action-plan pairs (rather than as plans only) means that it has some implicit robustness to missing actions. These action-plan pairs encode different lengths of suffixes and prefixes for a given plan. Shorter prefixes are more likely to match to **o** than longer ones are as there are less parts of the plan that need to match with **o**. Therefore, even when an action is missing in **o**, there will be rules in *E* that match to the correct goal that may collectively still lead Elexir-MCTS to return the correct answer.

To illustrate further, suppose **o** contains harvesting *two* wheat and gathering *one* wheat (i.e., it is missing the gathering action for the second wheat). This will not match with a rule for obtain bread where the plan prefix is to harvest and gather *two* wheat [i.e., **o** is **harvest(wheat), gather(wheat), harvest(wheat)** and plan-prefix of rule requires **harvest(wheat), gather(wheat), harvest(wheat), gather(wheat)**]. However, it will match to a rule obtain bread where the plan prefix is to harvest and gather *one* wheat [i.e., the prefix of rule is **harvest(wheat), gather(wheat)**]. Suppose instead that **o** contains harvesting and gathering both of the *two* wheat [i.e., **o** is **harvest(wheat), gather(wheat), harvest(wheat), gather(wheat)]**. Then, both the above plan prefixes of the rule for obtain bread will be matched, giving obtain bread a higher weight than if the second gathering action were missing.

Similar to PANDA-Rec, it is possible for Elexir-MCTS to fail to recognize a goal if the parameters of an action in **o** are different than expected. For each action in **o**, the types of the action's parameters must match the types of its corresponding action in the CCG; Elexir-MCTS will fail to recognize a goal if there is a type mismatch.

### 3.3. Refinement via Analogy for Goal Reasoning

Refinement via Analogy for Goal Reasoning (RAGeR) solves the goal recognition problem using analogical reasoning. RAGeR is based on the Analogical Theory of Mind (AToM) model (Rabkina et al., 2017). AToM is primarily a computational cognitive model of children's Theory of Mind (ToM) reasoning (Rabkina et al., 2017, 2018). AToM learns to reason about the mental states of others by analogically aligning an ongoing situation with previously-encountered stories or scenarios and making inferences based on overlapping structure (see below). For example, if it has previously seen that people expect cookie boxes to have cookies inside, AToM can infer that a person who sees a shoe box will expect it to have shoes inside. Similarly, AToM can make inferences about agents' goals based on prior observations (Rabkina and Forbus, 2019; Rabkina et al., 2020). However, AToM must be trained before it can perform goal recognition and, because it compares entire scenarios, cannot leverage the hierarchical knowledge available in preexisting domain models. RAGeR solves both of these problems, while maintaining AToM's robust inference capabilities.

For RAGeR, the goal recognition problem (*D*, **o**, *s*_0_, *G*) is translated into the 3-tuple (o,L,G) (the initial state *s*_0_ is not needed). The main difference is representational, as RAGeR uses a form of analogical reasoning that compares two cases—sets of logical expressions written in predicate calculus. Hence, **o** must be represented as a case, and *D* as a set of cases, called a case library.

When **o** is represented as a case, each individual observation is a single logical expression. The set of all expressions representing all the observations in **o** form a single case. Because this is not, in principle, different from the typical **o**, we keep the notation the same.

**Figure 1:**
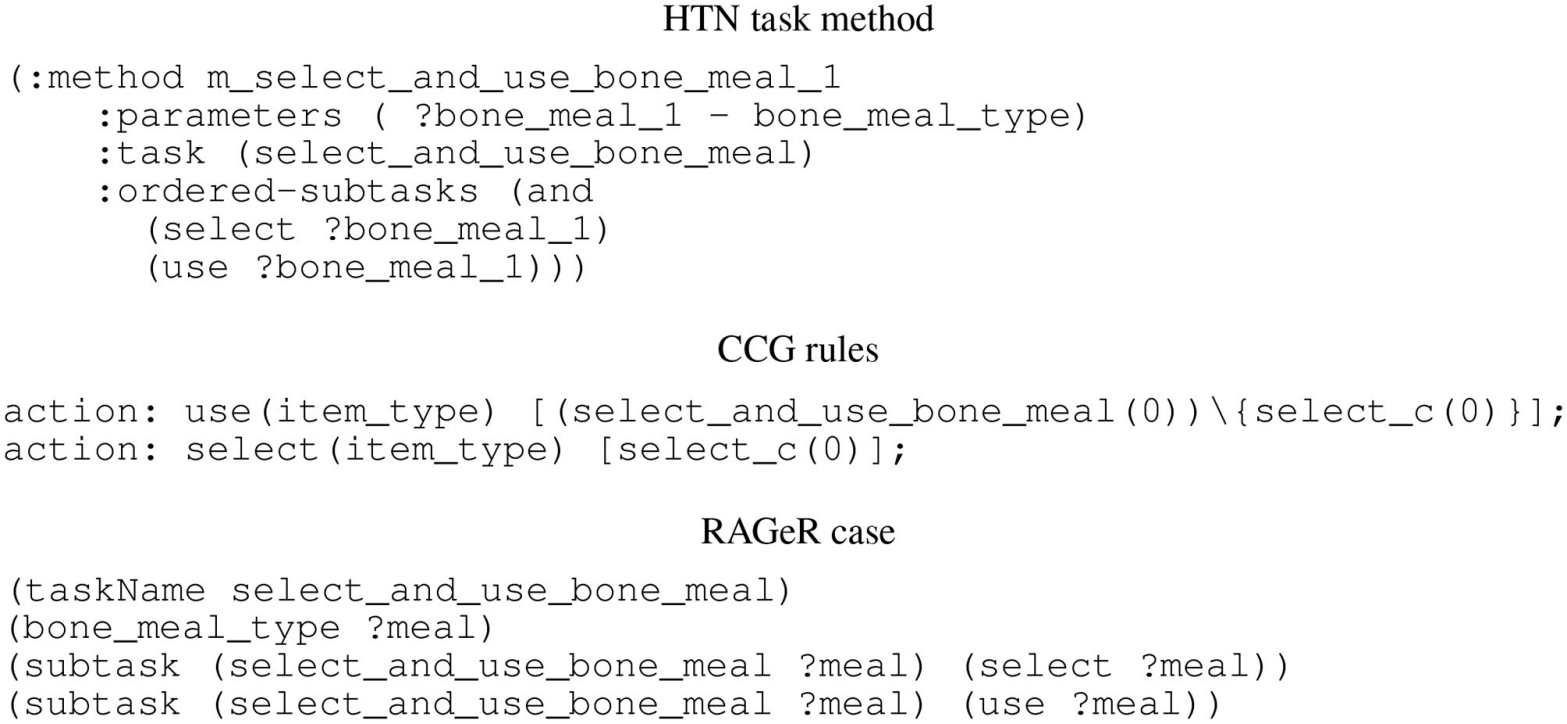
A method in the HTN model used by PANDA-REC, and corresponding CCG rules used by Elexir-MCTS and case in the case library used by RAGeR.

The set of cases L, on the other hand, is somewhat different from *D*. Assuming that *D* is an HTN (as is the case in the present experiments), each possible task decomposition must be converted into a case. Each decomposition consists of a parent task, some set of parameters, and a sequence of subtasks (see Figure 1). Each parameter is converted to an expression of the form **parameter_type(parameter_name)**. Additionally, the parameters are used to construct an expression relating the task name to the list of parameters [e.g., **select_and_use_bone_meal(?meal)**]. The subtasks are represented in the case as a set of statements that relate the task to one of the subtasks. Once the observations **o** and domain model *D* are represented as cases for **o** and L, the RAGeR algorithm is ready to begin the goal recognition process.

The RAGeR algorithm details are shown in Algorithm 1. At a high level, to infer the goal of an agent, RAGeR iteratively refines the **o** until it is abstracted up to a set of expressions that includes a goal. This is done by repeatedly replacing a subset of **o** with its corresponding parent task, which is found using analogical reasoning. Once the refined **o** contains a goal *g* ∈ *G*, the process is considered complete, and *g* is inferred to be the agent's goal. The process is analogous to walking up the HTN until the top of the tree-like structure is found. The key novelty in the RAGeR algorithm is in how, in the refinement process, it uses analogical reasoning, based on the AToM model, to find cases in the case library that correspond to parent tasks whose children are present in **o**.

**Algorithm 1:**
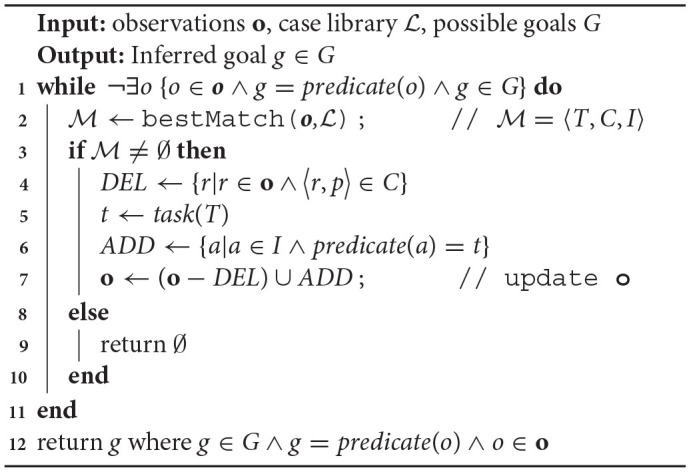
RAGeR Algorithm

Key to the refinement process is the retrieval of a case, from the case library L, that is similar to the current observation case **o**. Since the cases in the case library are task decompositions, RAGeR is effectively looking for the task decomposition that is most similar to the current set of observations. It then uses this task decomposition to update **o** and repeats the process until a goal is found.

**Figure 2:**
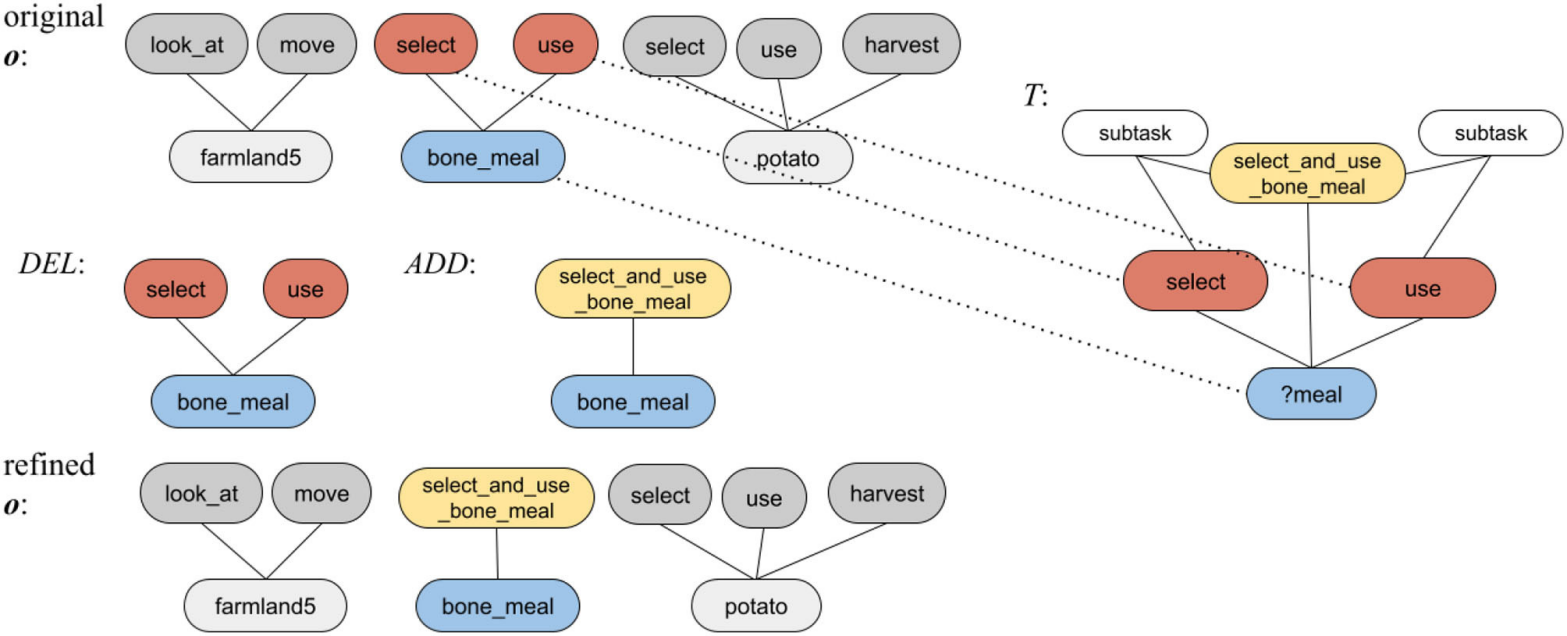
An illustration of RAGeR recognizing the subtask *select and use bone meal* in an observation of an agent completing an *obtain potato* task. The original **o** is the sequence of observations [**look_at(farmland5)**, **move(farmland5)**, **select(bone_meal)**, **use(bone_meal)**, **select(potato)**, **use(potato)**, **harvest(potato)**], *T* is the retrieved case, *DEL* and *ADD* are the expressions being used to update **o**, and the refined **o**′ is the updated observations, which are be used in the next round of refinement.

The process of finding the best decomposition case to **o**, designated in the RAGeR algorithm as bestMatch, uses analogical retrieval (Forbus et al., 1995) to search the case library for the most similar case in L (i.e., the best match). Similarity is defined as analogical similarity—overlap in the structure of two cases (see Figure 2).

To make this comparison, RAGeR uses the Structure Mapping Engine (SME; Forbus et al., 2017). SME determines the degree of analogical similarity and produces a similarity score, which is used as a metric during retrieval. SME also produces sets of correspondences and candidate inferences (described below). Together, the best matching case in the case library, *T*, the set of correspondences, *C*, and the set of candidate inferences, *I*, form the result of the bestMatch function. If no case in the case library is sufficiently similar to **o**, bestMatch returns null.

More concretely, when subtasks of a method are represented as a graph, as they are at the top right of Figure 2, the graph is typically connected via shared arguments. The actions comprising **o** can similarly be represented as a graph. These graph structures are the basis of the analogical comparison—similar structures in the graphs imply analogical similarity. SME identifies such corresponding structures between two cases. The pair 〈*r, p*〉 is a *correspondence*, where *r* is an element of the retrieved case and *p* is an element of the probe. The dashed lines in Figure 2 represent the correspondences in this example. Due to a *one-to-one correspondence* constraint (Gentner, 1983; Forbus et al., 2017), each element (whether entity or predicate) has at most one element in the other case with which in can be in correspondence.

SME also produces a set of *candidate inferences*, which are projections from the retrieved case onto the probe. In other words, they are parts of the retrieved case that correspondences suggest should be present in the probe, but are not. Candidate inferences include at least one element that is part of a correspondence. In the example in Figure 2, select_and_use_bone_meal is in the retrieved case but not in the probe. In the retrieved case, this element is part of the expression **select_and_use_bone_meal(?meal)**. Since **?meal** corresponds with **meal** in the probe, the expression **select_and_use_bone_meal(meal)** is supported by this correspondence and is thus a candidate inference.

RAGeR uses the bestMatch function to retrieve the case from the case library that is most similar to the current **o**. If bestMatch is successful, it returns M=〈T,C,I〉, where *T* is the retrieved case, *C* is the set of correspondences, and *I* is the set of candidate inferences. Given this match, RAGeR refines **o** by deleting the lower-level entries and replacing them with their parent expression. The set of expressions to be deleted are all of those that are in **o** and have a corresponding expression in the retrieved case *T*. In the example in Figure 2, the expressions **select(bone_meal)** and **use(bone_meal)** are to be removed from **o**. The deleted expressions are replaced by one expression representing the task described in the retrieved case. To identify this expression, RAGeR considers the expressions in *I* and selects the one where the predicate of the expression is the name of the task (identified by the taskName relation in the case). In our example, the expression **select_and_use_bone_meal(meal)** is a candidate inference, and it can be added to **o** because **select_and_use_bone_meal** is the task name.

RAGeR repeats this process of refining **o** until it contains a task that is a goal. When a goal *g* ∈ *G* is found to be in **o**, RAGeR returns *g*. RAGeR also has a configurable maximum number of iterations to prevent infinite looping and to constrain reasoning time.

## 4. Experiment Setup

To explore and analyze the relative strengths and weaknesses of the approaches, we conducted a series of experiments that focused on comparing and contrasting the three goal recognition approaches along two different axes: the *reliability* of observations, and the *inspectability* of the other agent's mental model. We evaluated the approaches in two different domains: the open-world computer game Minecraft, where the goal recognition approaches observed an agent procuring different food items, and the disaster management domain Monroe, where the goal recognition approaches observed an agent performing various disaster relief tasks.

### 4.1. Data Observability

#### 4.1.1. Inspectability

We compare the performance of the approaches with data representing three levels of inspectability: high, medium and low. The level of inspectability relates to the availability of information pertaining to the agent's mental state. At a high level, high inspectability means having access to the mental representations the agent uses to execute the plan. Low inspectability, on the other hand, limits the information to what a third agent can observe (i.e., no access to the performing agent's mental state). Medium inspectability provides direct access to an agent's intended executed actions, but not the reasons behind them.

**Figure 3:**
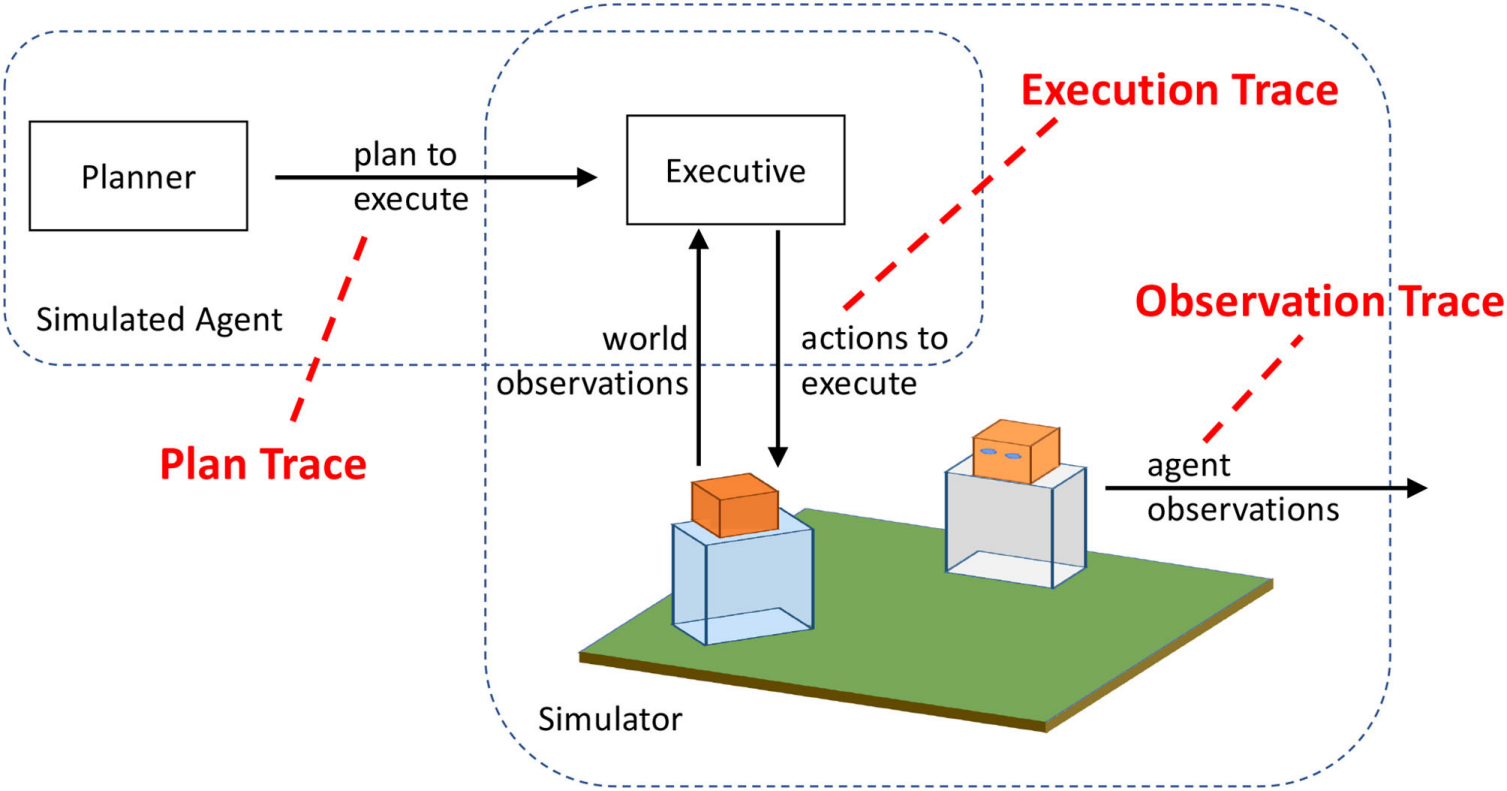
Three types of traces provide three levels of inspectability.

Practically speaking, each type of data arises from accessing an agent's mental “architecture” (whether real or artificial) in a different place (Figure 3):

- **High Inspectability**: Data for high inspectability comes from the *plan traces*, which are the actions that an agent plans to execute before execution begins. High inspectability is the result of having direct knowledge of what the agent intends to do, given what it knows about the world, to accomplish some unknown goal. This includes both actions and the arguments of those actions (such as what target an agent is walking to). In our experiments, a plan trace is the output of the agent's planner. Plan traces are commonly used in plan and goal recognition. For example, Sohrabi et al. (2016) generate optimal and suboptimal plans to test their plan recognition approach. Similarly, Blaylock and Allen (2006) use plan traces from the Monroe domain (see description below) for goal recognition. While the domain model used to generate these plans is not directly used in any of our goal recognition approaches, some knowledge of how the domain operates must be present in both the agents planner as well as the goal recognition models.
- **Medium Inspectability**: Data for medium inspectability comes from the *execution traces*, which are the actions that an agent executes in the world during execution. When an agent goes to execute a plan, the world may not be exactly as expected (changes in the world) and some actions may not perform exactly as intended (errors). As a result, the sequence of actions that the agent executes deviates from the ideal sequence of actions in the plan trace. The execution trace, which is a record of the agent's actions as it interacts with the world (simulated or real), may have actions skipped or repeated, and the parameters to the actions might slightly differ (e.g., slightly different coordinates). Execution traces can be captured from real or simulated world interactions. Traces recording the behaviors of game-playing agents in Real-Time Strategy games have been used to recognize agents' goals (Kantharaju et al., 2019). Others have generated synthetic traces using a stochastic simulator in constrained domains (Ramírez and Geffner, 2009) and goal-directed agent in an open-world simulated environment (Rabkina et al., 2020). The execution traces can provide some internal knowledge of the agent, reflected in the parameters of each action. Since the environment in which the agent is performing the actions is recording the actions, the intended target of the action is clear. This can be reflected in the parameters of actions, creating consistency in names of objects that is only possible through this greater access.
- **Low Inspectability**: Data for low inspectability comes from the *observation traces*, which is what an external agent can observe about an agent's behavior. Low inspectability means there is limited knowledge about the agent, since the actions that are recorded are from the perspective of another agent in the world. In many real-world scenarios, an observer will only have a partial view of states, actions, and effects, and recognizing goals from observation traces is necessary (Borrajo and Veloso, 2020). For example, an observing agent can see what object is in the performing agent's hand when it does an action with the object. However, the observing agent may not be able to discern the destination of a move action, as there are many ways to characterize any given location.

#### 4.1.2. Reliability

We examine two ways in which an observation may be more or less reliable: missing actions and incorrect parameters to an action. *Missing actions* are actions that are necessary for a goal to be achieved but are missing from the list of observed actions. A common way for missing actions to naturally appear in datasets is those that happen incidentally while accomplishing other actions. For example, in Minecraft (see section 4.2.1), an agent automatically picks up items as they walk by them, which can lead to missing actions when an agent incidentally already has completed an action that is required. Also, an observer may get to see only some of the actions an agent performs, making the agent's behavior partially observable. One approach to constructing synthetic data to measure the effects of missing observations is to filter the observations, often with three levels of filtering (Ramírez and Geffner, 2009; Sohrabi et al., 2016). We instead remove actions probabilistically (see sections 6 and 7).

Alternatively, observations can be *incorrect*, possibly due to sensor or judgment errors. One type of incorrect observation is to perceive the agent as doing a different action (Zhuo and Kambhampati, 2013). A more likely scenario may be that the parameters to an action may be incorrect. For example, an agent could be gathering carrots but the observation suggests the agent is gathering potatoes. The notion of unreliable observations due to incorrect parameters is somewhat similar to the definition of “noisy” observations from Sohrabi et al. (2016), who describe an observation as being noisy if there are state changes unrelated to the actions being performed. An agent holding potatoes would be construed to be a noisy observation if the agent was actually gathering carrots. We use this definition of incorrect observations here.

### 4.2. Datasets

#### 4.2.1. Minecraft

The first domain we consider is the open-world sandbox computer game Minecraft. Minecraft has been shown to be an interesting and challenging domain for studying many facets of AI (Johnson et al., 2016), and is particularly appropriate for studying goal recognition because (1) the set of possible goals an agent can have is open-ended and (2) because there are many flexible plans that can be constructed to achieve any given goal. For our experiments, we use the three Minecraft datasets introduced by Rabkina et al. (2020), where a Minecraft agent has one of seven top-level goals to procure food: obtain bread, obtain beef, obtain chicken meat, obtain potato, obtain pumpkin pie, obtain cake, and obtain carrot.

Table 1 shows metrics and measures of these three datasets. The first dataset consists of 100 high inspectability traces which contain the agent's mental deliberation on how it intends to achieve these goals. This mental deliberation takes the form of planning out the necessary sequence of actions to achieve these goals in ideal conditions. For example, to obtain pumpkin pie, an agent has to individually gather an egg, a bucket of milk, and a pumpkin that it finds in the environment. To obtain potato, an agent must first gather and then use a potato sprout and bonemeal, which is used during farming to speed up growth. When the potato has grown, the agent must harvest and then gather it. Similarly, obtaining chicken or beef involves attacking the animal and then gathering its meat. To obtain bread, an agent requires three wheat, which means gathering three wheat seeds, using them so they grow, then harvesting and gathering the wheat. Many of these actions also require the agent to move to the appropriate place to execute them, such as moving to a wheat seed it has found to collect it. Each instance in the dataset, then, is a plan that is constructed using an HTN planner paired with its corresponding top-level goal. Here, planning is done over the above seven top-level goals and 16 actions (e.g., **craft-item**, **move-near-target**, **look-at-entity**, **gather**, etc.).

**Table 1:** Metrics and measures for the three Minecraft datasets used in our experiments.

**Dataset**	**Minecraft high inspectability**							
**Goal**	**Obtain pumpkin** **pie**	**Obtain** **bread**	**Obtain** **potato**	**Obtain chicken** **meat**	**Obtain** **beef**	**Obtain** **cake**	**Obtain** **carrot**	**All**
Number of plans	5	18	22	13	35	3	4	100
Average plan length	5.00	37.0	19.0	5.00	5.00	47.0	19.0	19.6
**Goal**	**Obtain pumpkin** **pie**	**Obtain** **bread**	**Obtain** **potato**	**Obtain chicken** **meat**	**Obtain** **beef**	**Obtain** **cake**	-	**All**
**Dataset**	**Minecraft medium inspectability**							
Number of plans	33	101	201	154	360	43	-	892
Average plan length	2.30	16.7	6.54	3.75	3.76	14.6	-	7.94
**Dataset**	**Minecraft low inspectability**							
Number of plans	33	101	201	154	360	43	-	892
Average plan length	2.30	16.7	6.54	3.75	3.76	14.6	-	7.94

Once a plan has been developed, the agent attempts to achieve the plan by taking some sequence of observable actions in the game environment. The second dataset captures 849 of these medium inpsectable sequences of actions paired with their top-level goals. Here, the agent takes actions drawn from a set of seven actions: **gather**, **select**, **move**, **look at**, **attack**, **harvest**, and **use**.

There are three noteworthy features of this dataset to discuss. First, because of their relative values in Minecraft, the agent does not pursue goals with equal likelihood, so we see an imbalance in the types of possible goals. For example, obtain beef results in a large number of Minecraft “food points” and so it is the most frequently pursued goal. This corresponds to real-life situations, where people pursue goals with different frequencies. Second, as shown in Table 1, the goal obtain carrot is not found in this second dataset, but is found in the first dataset. This occurs if an agent plans for a goal and later discards it if a situation arises where a better goal can be pursued. This illustrates one key difference between deliberation and execution; while the agent can plan to do something, they may not actually do it.

Third, this dataset is unreliable in that missing actions can occur with some frequency in this dataset because an agent sometimes accomplishes the purpose of an action by pure luck, without explicitly executing it. For example, an agent needs to **move** near a chicken or cow before **attacking** it. However, when the agent is already next to a chicken or cow, it skips the **move** step. Another example occurs with **gather**, which only requires the agent to be near an item. An agent can, for instance, accidentally gather an egg while pursuing the obtain chicken meat goal. It may later use the egg to make a pumpkin pie, obviating the **gather** action for the egg. Another common cause of missing *gathering* actions is when an agent is close enough to plant that they are *harvesting* that they *gather* it without trying to. These examples, while situated in the Minecraft domain, have many analogues in real-life situations where goal recognition may be desirable.

The final dataset is a modification of the second dataset to have low inspectability. Specifically, the key changes made to this dataset were related to movement (i.e., **move**) and perceptual actions (i.e., **look at**). Both of these actions are applied over specific objects in the Minecraft environment, such as a particular chicken or cow. However, these specific objects in which the agent acts over is internal to the agent; we would not know if an agent moved to a specific chicken if we saw them move to a group of chickens. As such, we generalized these actions by adjusting their parameters such that the actions is applied over general locations than specific objects (i.e., movement to a chicken is translated into movement to a location within the chicken's vicinity).

#### 4.2.2. Monroe

The Monroe domain captures plans for disaster relief, including providing medical attention, plowing snow, and clearing debris from roads, and has been used in past plan and goal recognition work (Bisson et al., 2015). This domain was developed as part of a larger framework for generating artificial datasets for machine learning research, and for benchmarking plan and goal recognition systems (Blaylock and Allen, 2005). As such, we also use the Monroe domain for evaluating and comparing Elexir-MCTS, PANDA-Rec, and RAGeR.

The Monroe domain contains 10 top-level goals and 30 actions **navigate-snowplow**, **hook-up**, **clean-hazard**, etc. This is in contrast to the Minecraft domain, which contains 6-7 top-level goals and 16 (high inspectability dataset) and 7 (medium and low inspectability) actions. For our experiments, we used the first 100 plans from the publicly-available *monroe5000* dataset (Blaylock and Allen, 2005)^2^. Table 2 provides the distribution of the plans in the dataset and the average plan length for each goal. Compared to the Minecraft datasets, this Monroe dataset is more similar to the high inspectability dataset than the medium or low inspectability as it (1) does not contain missing actions due to execution (i.e., dataset is reliable) and (2) the plans in the dataset represent deliberation to provide disaster relief.

**Table 2:** Metrics and measures for the Monroe dataset used in our experiments.

**Dataset**	**Monroe**										
**Goal**	**Set up** **shelter**	**Fix water** **main**	**Clear road** **hazard**	**Clear road** **wreck**	**Plow road**	**Provide medical** **attention**	**Provide temp** **heat**	**Quell riot**	**Fix power** **line**	**Clear road** **tree**	**All**
Number of plans	6	2	9	19	21	11	10	6	9	7	100
Average plan length	12.3	12.5	12.3	10.5	7.67	4.82	20.8	6.83	10.8	14.9	11.3

### 4.3. Focus of Study

**Figure 4:**
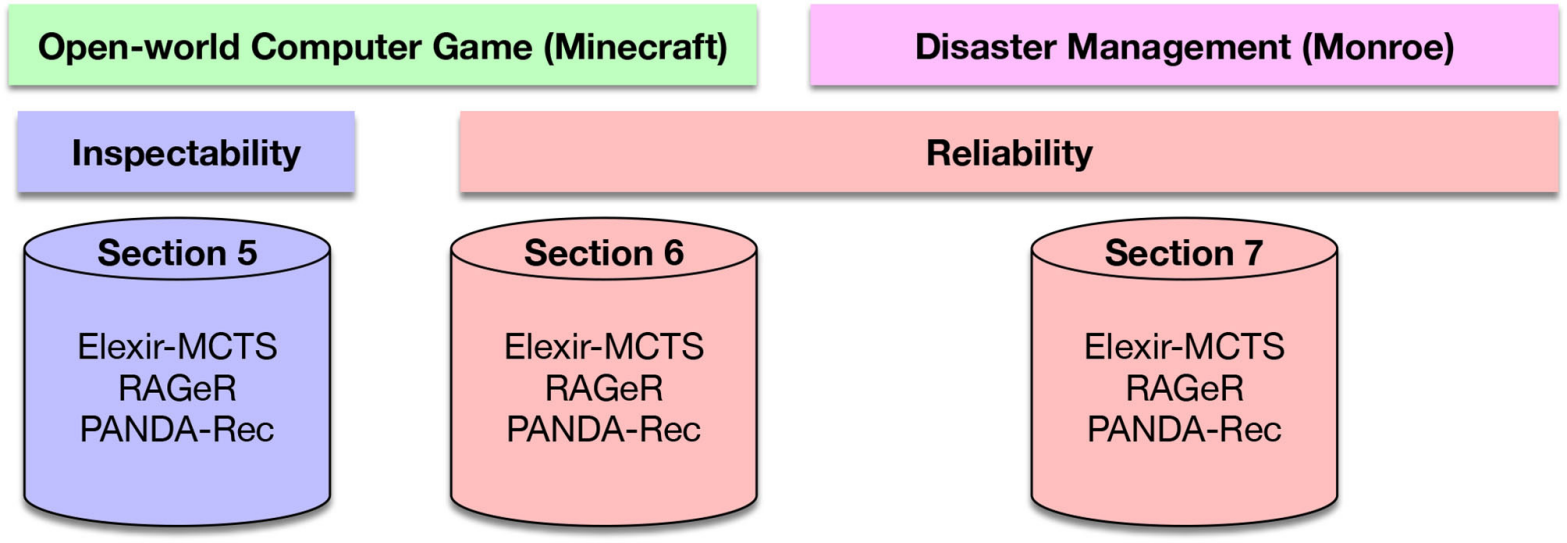
Diagram of study presented in paper. We evaluate inspectability of observations provided to Elexir-MCTS, RAGeR, and PANDA-Rec on data from the open-world computer game Minecraft in section 5. We then evaluate reliability of observations provided to Elexir-MCTS, RAGeR, and PANDA-Rec on data from Minecraft in section 6. We finally look at reliability of observations provided to Elexir-MCTS, RAGeR, and PANDA-Rec on data from the disaster management domain Monroe in section 7.

To analyze the relative strengths and weaknesses of the approaches, we conducted three experiments on two evaluation axes: inspectability and reliability. Figure 4 provides an overview of our experiments based on these axes.

**Experiment 1—Section 5:** The first experiment seeks to understand the performance of the three goal recognition systems presented in section 3 while exploring the axis of inspectability. Specifically, each approach is analyzed on the three Minecraft datasets presented in section 4.2.1.

**Experiment 2—Section 6:** The second experiment focuses on examining how sensitive each goal recognition approach is to unreliable actions from the Minecraft domain. We look at two different variants of unreliability over the actions: missing actions and incorrect action parameters. We model these variants of unreliability and apply them to the medium inspectability Minecraft dataset presented in section 4.2.1.

**Experiment 3—Section 7:** The third and final experiment aims to evaluate the goal recognition methods on unreliable actions from the Monroe domain. Similar to the second experiment, we look at two variants of unreliability: missing actions and incorrect action parameters. We model these variants of unreliability and apply them to the Monroe dataset presented in section 4.2.2.

## 5. Experiment 1: Evaluating Hierarchical Recognition While Varying Inspectabiliy

First, we evaluate PANDA-Rec, Elexir-MCTS, and RAGeR on goal recognition in the Minecraft domain with varying levels of agent inspectability^3^. Recall that high inspectability data are plans sent from the planner to the executive, medium inspectability data are action execution instructions sent from the executive to the agent, and low inspectability data are observations that are available to a third party, and are generated by removing knowledge that would only be available to the agent from medium inspectability traces.

For this experiment, we constructed an HTN and a CCG representing the goals and actions in the Minecraft domain (for PANDA-Rec and Elexir-MCTS, respectively), as well as a corresponding analogy case library for RAGeR. The HTN and CCG were hand-authored by one of the authors with knowledge about the Minecraft domain. We ensured that the HTN and CCG capture the same knowledge to the best of our ability. The analogy case library used by RAGeR was semi-automatically^4^ generated from the HTN model for consistency.

We also include two random baselines for each experimental condition. The first is a uniform random baseline; it generated its interpretation of the agent's goal by sampling uniformly across goals that appear in the data set. The second is a biased random baseline, with each potential goal weighted by its prevalence in the data set.

### 5.1. Inspectability: High

We begin by evaluating PANDA-Rec, Elexir-MCTS, and RAGeR on recognition of goals when the available traces are from the agent's internal planner. Precision, recall, and F1 scores for each algorithm can be found in Table 3. As the table shows, PANDA-Rec performs perfectly on this dataset, with 100% on all metrics. Elexir-MCTS and RAGeR also perform well, but do not match PANDA's performance, with F1 scores of 0.857 and 0.824, respectively.

**Table 3:** Macro Precision, Recall, and F1 scores for Elexir-MCTS, PANDA-Rec, RAGeR, and two baselines (Random Uniform and Biased recognition).

	**High inspectability**			**Medium inspectability**			**Low inspectability**		
	**Precision**	**Recall**	**F1 score**	**Precision**	**Recall**	**F1 score**	**Precision**	**Recall**	**F1 score**
Elexir-MCTS	**1.000**	0.810	0.857	**0.965**	0.780	**0.852**	**0.965**	**0.780**	**0.852**
PANDA-Rec	**1.000**	**1.000**	**1.000**	0.801	0.678	0.727	0.801	0.678	0.727
RAGeR	0.804	0.857	0.824	0.841	**0.840**	0.806	0.814	0.714	0.651
Uniform	0.131	0.139	0.111	0.162	0.168	0.143	0.162	0.168	0.143
Biased	0.151	0.146	0.145	0.169	0.169	0.169	0.169	0.169	0.169

*Macro values are averaged over 10 runs for Uniform and Biased. Bold values indicate the best performance in each condition*.

**Figure 5:**
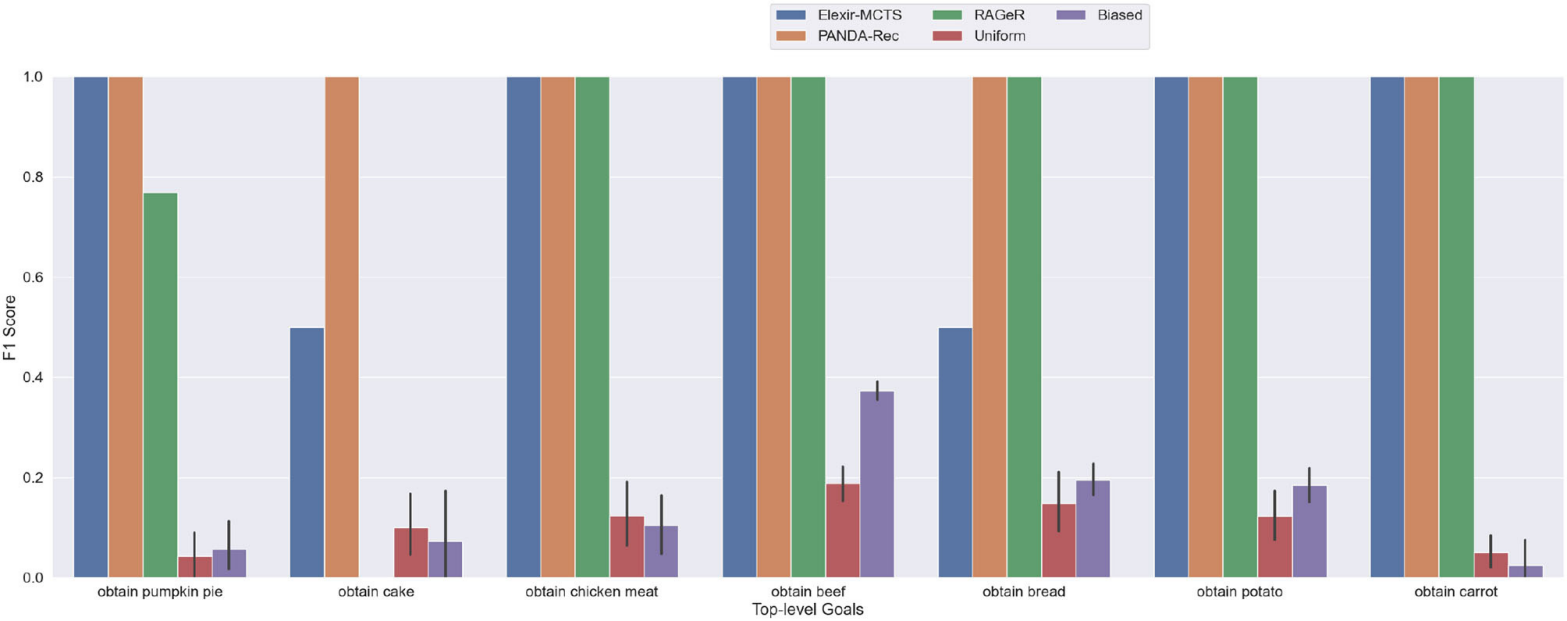
Performance of PANDA-Rec, Elexir-MCTS, RAGeR, and two baselines (Random Uniform and Random Biased Recognition) over the **high-inspectability Minecraft traces**. Metric shown in the figure is the F1 score for each top-level goal in the Minecraft domain. Random Uniform and Random Biased bars are calculated for each top-level goal by taking the average F1 score over 10 runs.

Figure 5 shows F1 scores for individual top-level goals. All three algorithms were able to recognize obtain chicken meat, obtain beef, obtain potato, and obtain carrot perfectly. Interestingly, the obtain cake goal accounts for most of the errors for both Elexir-MCTS and RAGeR—Elexir-MCTS was unable to recognize several of the obtain cake plans within the provided time limit, while RAGeR always confused it for obtain pumpkin pie. While obtain cake was the goal for only 3 cases in this dataset, it is clear that recognizing it is more difficult than the others. Notably, the average number of actions in a plan for obtain cake was 47, in contrast to, for example, 5 for obtain beef (Table 1). Furthermore, the models for obtain cake share many actions and parameters with both obtain bread and obtain pumpkin pie, making it the most complicated goal in the data set.

### 5.2. Inspectability: Medium

In the high inspectability condition, the algorithms had access to plan traces, which provide perfect information about the plans being executed by an agent. However, the execution of a plan is rarely perfect, and may not correspond exactly to a plan. As such, we next evaluate PANDA-Rec, Elexir-MCTS, and RAGeR on recognition of goals using the agent's report of its executed actions. All results are reported in Table 3. As before, PANDA-Rec, Elexir-MCTS, and RAGeR were provided models corresponding to the executed actions.

**Figure 6:**
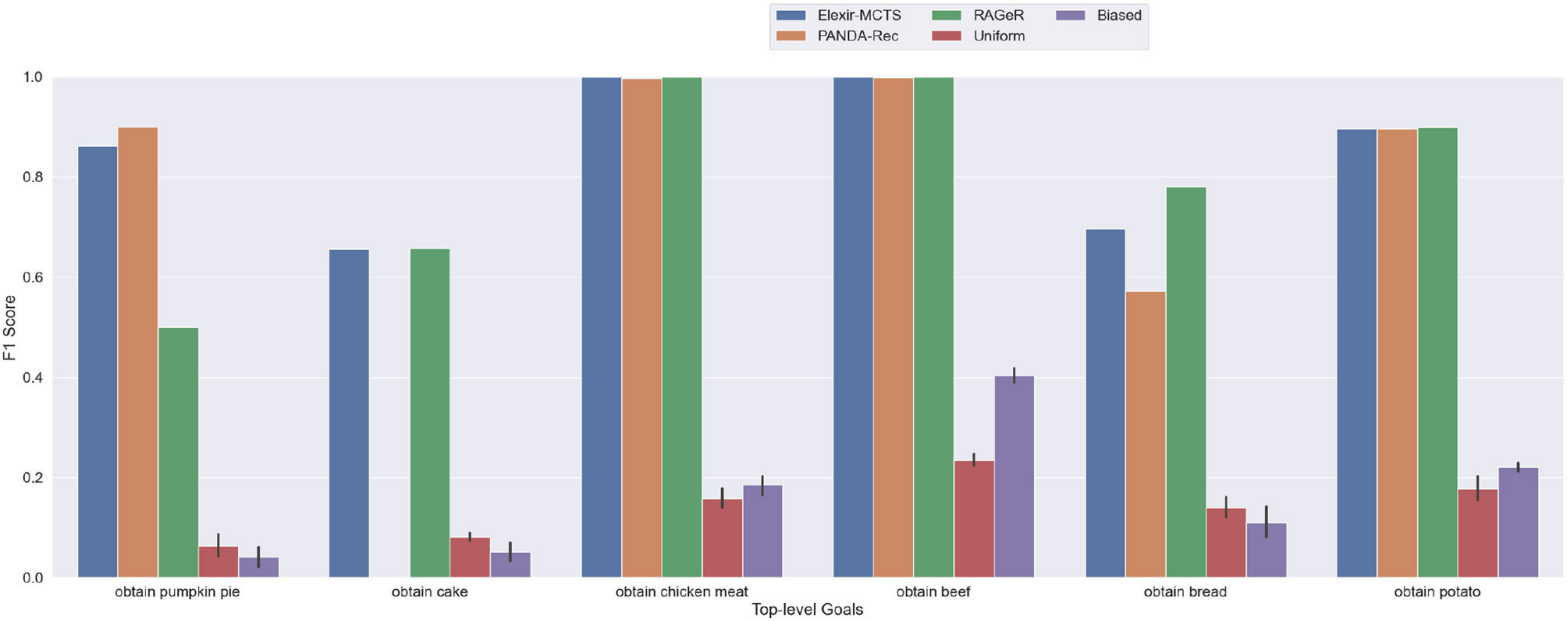
Performance of PANDA-Rec, Elexir-MCTS, RAGeR, and two baselines (Random Uniform and Random Biased Recognition) over the **medium-inspectability Minecraft traces**. Metric shown in the figure is the F1 score for each top-level goal in the Minecraft domain. Random Uniform and Random Biased bars are calculated for each top-level goal by taking the average F1 score over 10 runs.

PANDA-Rec's performance decreased the most between the high and medium inspectability conditions, from 1.00 F1 score to 0.727. As shown in Figure 6, its performance on the obtain cake goal dropped to 0, and its scores on obtain pumpkin pie, obtain bread, and obtain potato also decreased. PANDA-Rec performed poorly on traces for the obtain cake goal because it either failed to recognize any goal from the traces or mistook obtain cake for obtain bread (both require harvesting and gathering three wheat). Elexir-MCTS, on the other hand, maintained performance with an aggregate F1 score of 0.852. Its distribution of errors shifted, however, with decreases in performance on obtain pumpkin pie and obtain potato, and increases for obtain cake and obtain bread. RAGeR's performance also decreased slightly, to an F1 score of 0.806. Much like Elexir-MCTS, RAGeR's F1 for obtain cake improved, while obtain pumpkin pie, obtain bread, and obtain potato decreased. All three continued to perform perfectly on obtain beef and obtain chicken meat goals.

### 5.3. Inspectability: Low

In many human-machine teaming scenarios, knowledge of an human's mental/internal state (including knowledge, beliefs, and desires—but also specific targets and intended outcomes of actions) is not observable by a third-party agent making inferences about goals. Instead, agents must reason based only on their own observations of compatriots' behavior without access to internal state information, such as what is available in our high and medium inspectability data sets. To mimic this reality, we evaluate the goal recognition algorithms' performance on low inspectability data, in which information that would not be available to an outside observer is removed.

**Figure 7:**
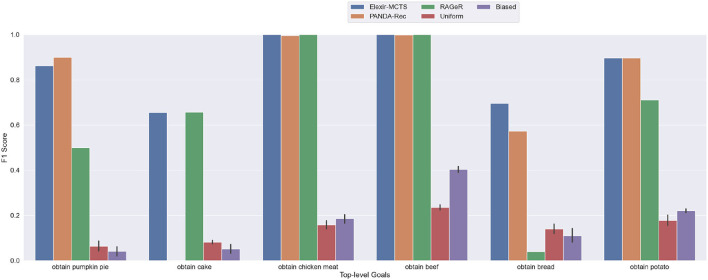
Performance of PANDA-Rec, Elexir-MCTS, RAGeR, and two baselines (Random Uniform and Random Biased Recognition) over the **low-inspectability Minecraft traces**. Metric shown in the figure is the F1 score for each top-level goal in the Minecraft domain. Random Uniform and Random Biased bars are calculated for each top-level goal by taking the average F1 score over 10 runs.

As shown in Table 3, Elexir-MCTS and PANDA-Rec's performance metrics did not change between the medium and low inspectability conditions. However, RAGeR performed worse, dropping in F1 score from 0.806 to 0.651. Figure 7 shows that, similar to the medium and high inspectability results, Elexir-MCTS, RAGeR, and PANDA-Rec have near perfect F1 scores on obtain chicken meat and obtain beef. Similar to the medium inspectability condition, PANDA-Rec has an F1 score of 0 for the obtain cake goal. In general, the biggest change between the medium- and low-inspectability condition was RAGeR's ability to recognize obtain bread, which dropped from an F1 score of 0.78 to 0.04. This was due to confusion with obtain potato, which had several shared subgoals.

## 6. Experiment 2: Evaluating the Impact of Missing Actions and Noisy Parameters

In this experiment, we take a closer look at the medium inspectability results from the first experiment. We introduce here a new variable: *reliability* of the data source. We compare the baseline result from experiment 1 (the *no noise* condition) with two ways in which unreliable sensor data may manifest itself: *missing actions* and *incorrect parameters* on actions. For each type of noise, we tested all three algorithms at 25, 50, and 75% percent noise.

To generate the incorrect parameters data sets, we modified the no noise baseline data set by randomly replacing parameters with objects in the domain, according to the noise percentage of the condition. More specifically, given a trace and a set of objects in the domain, each parameter of the actions was replaced with a random object with a probability corresponding to the noise percentage of the data set (i.e., in the 25% noise dataset, each parameter was replaced with a probability of 0.25). Parameters were replaced with an object sampled uniformly across all objects in the domain—including those shared across the data set (e.g., **iron-sword** or **air**) and those specific to a particular trace (e.g., **chicken34**).

The missing actions data sets were generated similarly. However, instead of randomly replacing parameters of actions in traces, we removed actions entirely. That is, each action in each trace was removed with a probability corresponding to the data set's noise percentage. This sometimes resulted in empty traces, as noted in the analyses below.

### 6.1. Reliability: Missing Actions

Elexir-MCTS, PANDA-Rec, and RAGeR's performance on data with missing actions can be found in Table 4. Elexir-MCTS and PANDA-Rec's performance decreased with missing actions. Without noise, Elexir-MCTS had an F1 score of 0.852. This dropped to 0.541 with 25% missing actions, and continued to drop to 0.368 at 50% and 0.196 at 75%. Similarly, PANDA-Rec had an F1 score of 0.727 in the original dataset, and fell to 0.459 at 25% missing actions, 0.376 at 50% and 0.315 at 75%. RAGeR, on the other hand, maintained performance, increasing slightly from 0.806 F1 without noise to 0.814 F1 with 25% probability of missing actions. Its F1 score continued to increase slightly to 0.821 at 50% missing actions and 0.831 at 75%. This counter intuitive improvement in performance, while slight, can be attributed to non-deterministic actions (i.e., those that are not discriminative between goals) being more likely to be removed (because there are more of them), and therefore leaving behind a stronger signal to noise ratio for RAGeR.

**Table 4:** Average macro Precision, Recall, and F1 scores for PANDA-Rec, Elexir-MCTS, RAGeR, and two baselines (Random Uniform and Random Biased Recognition) over unreliable traces from the Minecraft domain.

	**0.0%**			**25.0%**			**50.0%**			**75.0%**		
	**Precision**	**Recall**	**F1 score**	**Precision**	**Recall**	**F1 score**	**Precision**	**Recall**	**F1 score**	**Precision**	**Recall**	**F1 score**
**Reliability - Missing**												
Elexir-MCTS	**0.965**	0.780	**0.852**	**0.924**	0.451	0.541	0.798	0.281	0.368	0.576	0.129	0.196
PANDA-Rec	0.801	0.678	0.727	0.717	0.374	0.459	0.682	0.295	0.376	0.701	0.224	0.315
RAGeR	0.841	**0.840**	0.806	0.845	**0.845**	**0.814**	**0.849**	**0.847**	**0.821**	**0.859**	**0.849**	**0.831**
Uniform	0.162	0.168	0.143	0.162	0.168	0.143	0.162	0.168	0.143	0.162	0.168	0.143
Biased	0.169	0.169	0.169	0.169	0.169	0.169	0.169	0.169	0.169	0.169	0.169	0.169
**Reliability - Incorrect**												
Elexir-MCTS	**0.965**	0.780	**0.852**	0.803	0.457	0.518	0.640	0.342	0.387	0.486	0.254	0.287
PANDA-Rec	0.801	0.678	0.727	0.761	0.213	0.310	0.627	0.067	0.114	0.411	0.014	0.025
RAGeR	0.841	**0.840**	0.806	**0.819**	**0.807**	**0.777**	**0.791**	**0.745**	**0.705**	**0.784**	**0.709**	**0.665**
Uniform	0.162	0.168	0.143	0.162	0.168	0.143	0.162	0.168	0.143	0.162	0.168	0.143
Biased	0.169	0.169	0.169	0.169	0.169	0.169	0.169	0.169	0.169	0.169	0.169	0.169

*Each score was averaged over 10 runs (excluding 0% noise). Bold values indicate the best performance in each condition*.

**Figure 8:**
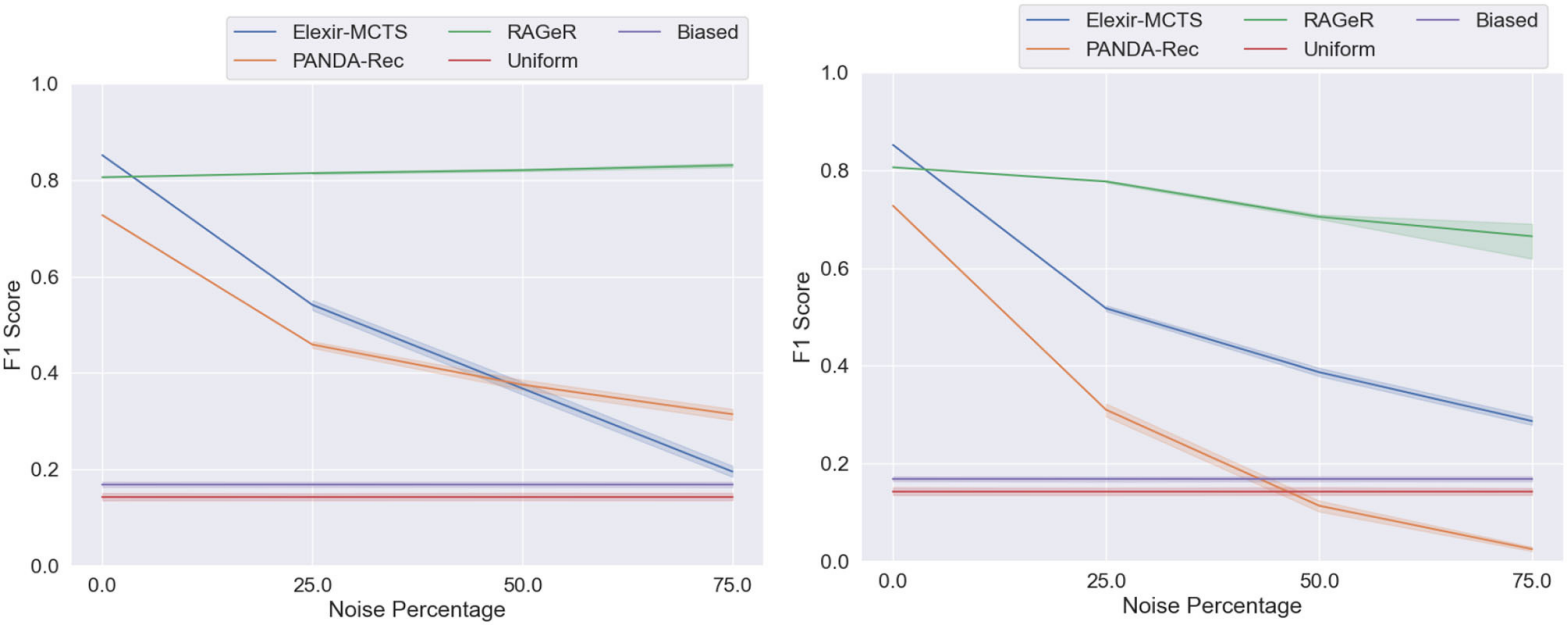
Performance of PANDA-Rec, Elexir-MCTS, RAGeR, and two baselines (Random Uniform and Random Biased Recognition) over unreliable traces from the Minecraft domain. Figures represent the performance of methods with regards to unreliability resulting from missing actions **(left)** and incorrect actions **(right)**. We varied the noise percentage from 0 to 75% in increments of 25%, and all methods were run for 10 runs for each noise percentage (excluding 0% noise). Each line represents a 95% confidence interval for the average macro F1 scores over the 10 runs.

To determine whether RAGeR, Elexir-MCTS, and PANDA-Rec differed in performance across noise levels, A 3 × 3 Two-Factor ANOVA with Repeated Measures was used^5^. It revealed a main effect of algorithm [*F*_(2, 18)_ = 6,639, *p* < 0.0001] and noise level [*F*_(2, 18)_ = 937, *p* < 0.0001]. A significant interaction between algorithm and noise level [*F*_(4, 36)_ = 823, *p* < 0.0001] was also found. As can be seen in Figure 8, while performance generally decreased as data became less reliable, RAGeR was not affected by missing actions, while than Elexir-MCTS and PANDA-Rec were.

### 6.2. Reliability: Incorrect Parameters

Table 4 shows how Elexir-MCTS, PANDA-Rec, and RAGeR's performance change as the reliability of the Minecraft data changes due to incorrect parameters. Similar to their performance on lowered reliability due to missing actions, Elexir-MCTS and PANDA-Rec steadily drop in performance as reliability decreases due to incorrect parameters, while RAGeR remains relatively stable. Unlike in the missing actions condition, howev, RAGeR's performance does decrease slightly, from an F1 score of 0.806 with no noise to 0.0.777 at 25% incorrect parameters, to 0.705 at 50% and 0.688 at 75%. PANDA-Rec's performance degrades more rapidly with incorrect parameters, falling from 0.727 with no noise to 0.026 at 75%. This is due to strict type checking in PANDA-Rec—it cannot match actions in an observation sequence whose parameters are not of the expected type found in the HTN.

Elexir-MCTS' performance degrades with more incorrect parameters, dropping from 0.852 with no noise to 0.287 at 75% noise. Similar to PANDA-Rec, Elexir-MCTS does not match actions in a sequence whose parameters are not of the expected type found in the CCG. However, Elexir-MCTS appears to be more robust to incorrect parameters than PANDA-Rec.

As before, A 3 × 3 Two-Factor ANOVA with Repeated Measures^5^ revealed a main effect of algorithm [*F*_(2, 18)_ = 369, *p* < 0.0001] and noise level [*F*_(2, 18)_ = 62, *p* < 0.0001]. However, no interaction was found between the two [*F*_(4, 36)_ = 2, *p* = 0.086]. This indicates that, while the algorithms differed in overall performance, all three tended to perform worse as reliability decreased and percent noise increased (Figure 8).

## 7. Experiment 3: Generalizing Results to Disaster Relief

Having used the Minecraft domain to examine how the various approaches are impacted by inspectability and reliability, we next test the approaches on a more complex domain. Like Minecraft, the Monroe disaster relief domain was crafted to test goal recognition systems. However, it has more top-level goals and actions than the Minecraft domain, making goal recognition more complicated. Thus, we use this data set to generalize our findings, focusing on data reliability.

For the below experiments, we used the first 100 traces from the *monroe5000* dataset (Blaylock and Allen, 2006). These are the equivalent of Minecraft high inspectability plans because they come directly from the Monroe planner; there is no simulator associated with this dataset. As with Experiment 2 above, we vary the reliability of the data source in two ways: by removing actions and adding incorrect parameters.

The HTN used by PANDA-Rec was the same as the one used by Höller et al. (2018)^6^. This model was manually converted to the CCG used by Elexir-MCTS by one of the authors. All effort was taken to ensure that knowledge remained consistent between the two models. The analogy case library used by RAGeR was semi-automatically generated from the HTN.

### 7.1. Monroe Reliability: Missing Actions

Table 5 shows the Precision, Recall, and F1 scores for Elexir-MCTS, PANDA-Rec, and RAGeR on the Monroe data set as reliability decreases. When no noise is added, Elexir-MCTS has the highest F1 score at 0.916. PANDA-Rec also performs well, with an F1 score of 0.880. RAGeR's performance is substantially worse, with a 0.408 F1 score at 0% noise. However, while PANDA-Rec and Elixir's performance drops as reliability decreases (to 0.274 and 0.228 F1 with 25% noise), RAGeR's remains at 0.406 F1.

**Table 5:** Average macro Precision, Recall, and F1 scores for PANDA-Rec, Elexir-MCTS, RAGeR, and two baselines (Random Uniform and Random Biased Recognition) over unreliable traces from the Monroe domain.

	**0.0%**			**25.0%**			**50.0%**			**75.0%**		
	**Precision**	**Recall**	**F1 score**	**Precision**	**Recall**	**F1 score**	**Precision**	**Recall**	**F1 score**	**Precision**	**Recall**	**F1 score**
**Reliability - Missing**												
Elexir-MCTS	0.946	**0.899**	**0.916**	**0.660**	0.197	0.274	0.377	0.075	0.104	0.126	0.027	0.031
PANDA-Rec	**1.000**	0.834	0.880	0.646	0.149	0.228	0.260	0.044	0.073	0.246	0.047	0.070
RAGeR	0.382	0.456	0.408	0.422	**0.451**	**0.406**	**0.498**	**0.388**	**0.386**	**0.449**	**0.248**	**0.270**
Uniform	0.135	0.142	0.123	0.135	0.142	0.123	0.135	0.142	0.123	0.135	0.142	0.123
Biased	0.078	0.081	0.078	0.078	0.081	0.078	0.078	0.081	0.078	0.078	0.081	0.078
**Reliability - Incorrect**												
Elexir-MCTS	0.946	**0.899**	**0.916**	0.130	0.018	0.030	0.010	0.001	0.002	0.000	0.000	0.000
PANDA-Rec	**1.000**	0.834	0.880	0.120	0.017	0.028	0.010	0.001	0.002	0.000	0.000	0.000
RAGeR	0.382	0.456	0.408	**0.348**	**0.424**	**0.338**	**0.310**	**0.363**	**0.285**	**0.261**	**0.296**	**0.234**
Uniform	0.108	0.118	0.100	0.108	0.118	0.100	0.108	0.118	0.100	0.108	0.118	0.100
Biased	0.083	0.084	0.082	0.083	0.084	0.082	0.083	0.084	0.082	0.083	0.084	0.082

*Each score was averaged over 5 (missing actions) or 10 (incorrect actions) runs (excluding 0% noise). Bold values indicate the best performance in each condition*.

**Figure 9:**
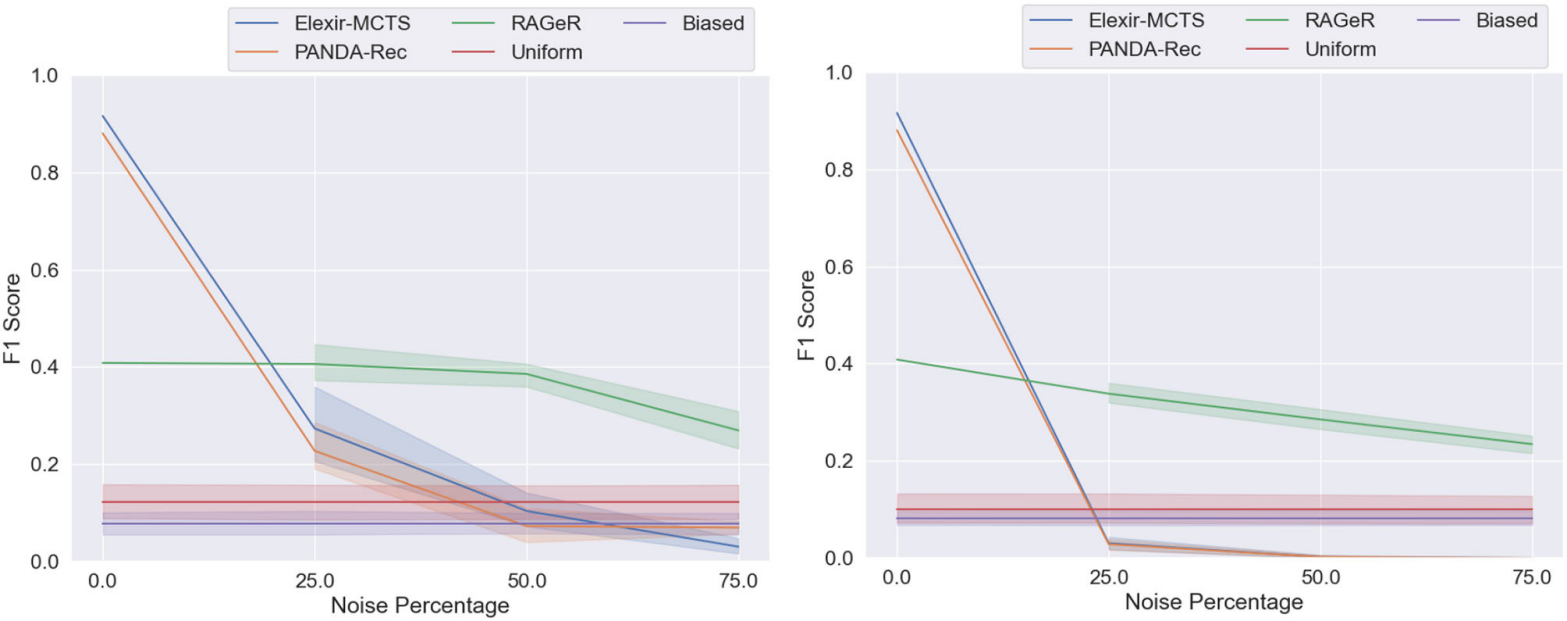
Performance of PANDA-Rec, Elexir-MCTS, RAGeR, and two baselines (Random Uniform and Random Biased Recognition) over unreliable traces from the Monroe domain. Figures represent performance of methods with regards to unreliability resulting from missing actions **(left)** and incorrect actions **(right)**. We varied the noise percentage from 0 to 75% in increments of 25%, and all methods were run for 5 (missing actions) or 10 (incorrect actions) runs for each noise percentage (excluding 0% noise). Each lines represent a 95% confidence interval for the average macro F1 scores over the 5 or 10 runs.

A 3 × 3 Two-Factor ANOVA with Repeated Measures^5^ revealed a main effect of algorithm [*F*_(2, 8)_ = 79, *p* < 0.0001] and noise level [*F*_(2, 8)_ = 46, *p* < 0.0001], as well as an interaction between the two [*F*_(4, 16)_ = 5, *p* = 0.0083]. As can be seen in Figure 9, this is consistent with the pattern of results when data reliability was decreased with missing actions in the Minecraft dataset in section 6: Elexir-MCTS and PANDA-Rec decrease in performance more rapidly than does RAGeR.

### 7.2. Monroe Reliability: Incorrect Parameters

Performance metrics for Elexir-MCTS, PANDA-Rec, and RAGeR as incorrect parameters are added to the Monroe domain can be found in Table 5. As with all previous noise conditions, RAGeR's scores do not change substantially from 0% noise to 75%. Elexir-MCTS and PANDA-Rec, however, drop below both random baselines with just 25% added noise (to F1 scores of 0.0030 and 0.028, respectively). At 75% noise, both Elexir-MCTS and PANDA-Rec have F1 scores of 0. As with the Minecraft domain, this poor performance is likely due to type checking: neither algorithm can continue with recognition when an action's argument with an incorrectly typed parameter is passed into them.

A 3 × 3 Two-Factor ANOVA with Repeated Measures^5^ revealed a main effect of algorithm [*F*_(2, 18)_ = 1,518, *p* < 0.0001] and noise level [*F*_(2, 18)_ = 38, *p* < 0.0001]. A significant interaction between algorithm and noise level [*F*_(4, 36)_ = 12, *p* < 0.0001] was also found. As can be seen in Figure 9, all three algorithms perform worse as data reliability decreases and noise is added. However, RAGeR's performance is much more consistent than Elexir-MCTS and PANDA-Rec under these conditions.

## 8. Discussion

We have tested three goal recognition algorithms on their ability to recognize goals under varying levels of inspectability and data reliability. The algorithms each take different approaches to solving goal recognition problems: Elexir-MCTS uses CCGs to recognize goals; PANDA-Rec turns goal recognition into a planning problem; and RAGeR performs repeated analogical retrievals to find appropriate goals. RAGeR is a novel algorithm, so we are particularly interested in how it compares to the more-established Elexir-MCTS and PANDA-Rec.

It is clear from the results of our experiments that each of the three algorithms has different strengths and weaknesses when solving goal recognition problems. In particular, PANDA-Rec performs perfectly on the high inspectability Minecraft data set. Neither of the other algorithms perform perfectly on any data set. However, PANDA-Rec's performance decreases faster than both Elexir-MCTS and RAGeR when the data is less reliable and inspectable.

Elexir-MCTS is more robust to the degradation of agent inspectability compared to PANDA-Rec and RAGeR. While its performance in the Minecraft high inspectability condition is not quite as high as PANDA-Rec's, its precision, recall, and F1 drop only slightly as agent inspectability decreases. It also has the highest performance on the fully-reliable Monroe dataset, with an F1 score of 0.916. Like PANDA-Rec, however, Elexir-MCTS is not robust to data reliability, and its performance drops rapidly in both the Minecraft and Monroe data sets as reliability decreases. In fact, both Elexir-MCTS and PANDA-Rec have 0 precision, recall, and F1 with 75% incorrect parameters on the Monroe data set. They perform only slightly better at 25 and 50% incorrect parameters, and across the missing actions conditions.

On the other hand, RAGeR is fairly robust to changes in data reliability compared to Elexir-MCTS and PANDA-Rec. Its performance does not change substantially actions are removed and incorrect parameters are inserted in the Minecraft data set. Similarly, it maintains performance well above chance on the low reliability Monroe data sets, even while Elexir-MCTS and PANDA-Rec drop to 0. Yet, RAGeR tops out at 0.408 F1 on the fully reliable Monroe data set (compared to 0.916 and 0.880 for Elexir-MCTS and PANDA-Rec, respectively).

One limitation of our findings is the differing goal recognition models given to each of Elexir-MCTS, PANDA-Rec, and RAGeR. While we did our best to keep the knowledge and representations uniform, differences inherent to the algorithms made direct transfer impossible: Elexir-MCTS needed a CCG, PANDA-Rec needed an HTN, and RAGeR needed an analogical case library. It is possible that, despite our best efforts, one algorithm or another had access to knowledge that the others did not have. Conversely, it is possible that one algorithm was able to make better use of available knowledge than the others simply because the model we created was a better fit. An interesting extension to this work that would mitigate any differences is to update models during run time to account for any discrepancies (e.g., Chakraborti et al., 2019).

What is clear from our findings is that Elexir-MCTS and PANDA-Rec have limitations when it comes to their robustness to data reliability, especially with respect to incorrect action parameters. These incorrect action parameters occur when an observing agent's perceptual systems fail or make mistakes (i.e., confusing a cup with a jar). While computer vision systems have seen drastic improvement over the years, they are still susceptible to errors and mistakes due to noise (Dodge and Karam, 2016). Given that Elexir-MCTS and PANDA-Rec rely on the correctness of the action's parameters for goal recognition, these perceptual errors would make it difficult or impossible for them to recognize goals. As such, these methods (and other goal recognition methods) should be extended to account for perceptual errors when recognizing goals.

RAGeR, on the other hand, is more robust to these kinds of errors. Yet, its baseline performance is lackluster, especially on the more complicated Monroe dataset. This is likely due to the fact that RAGeR is greedy: it always retrieves the case with the highest analogical match score, and never considers alternatives (even if their match score is equivalent or very close). On the other hand, Elexir-MCTS considers multiple possible goals before making a decision and PANDA-Rec generates multiple candidate plans during its search. Keeping parallel options available in this way keeps the algorithms from committing to incorrect paths too early, and allows them to prune as they go. Moving RAGeR to a Best First Search implementation should similarly add flexibility to its reasoning without sacrificing its major strength: robustness to unreliable data.

Given our results, we present a few suggestions on where the methods compared in this work could be applied. We note that these methods were evaluated on two domains in the present work, and we encourage future work to evaluate these methods on additional challenging domains. Our results suggest that PANDA-Rec is more applicable than Elexir-MCTS and RAGeR to high inspectability data, such as plan traces from an AI planner. This makes sense as PANDA-Rec utilizes an AI planner (specifically an HTN planner) in its reasoning. Elexir-MCTS is more appropriate than PANDA-Rec and RAGeR for medium to low inspectability data, such as traces from an agent's execution in an environment. Finally, RAGeR is much more appropriate than Elexir-MCTS and PANDA-Rec when there is unreliable data resulting from missing actions or incorrect action parameters.

We also suggest two interesting areas for future work in goal recognition. First, different variants of reliability should be explored. In particular, there may be other forms of noise than missing actions or incorrect action parameters that are encountered when attempting to recognize the plans of other agents in the real world. Second, it is important that a method of evaluating across different goal recognition approaches is developed. This work evaluated three fundamentally different methods for goal recognition, each with different model representations and algorithms. Having a method of evaluating across different goal recognition approaches that takes into account the differences in the methods would enable a much broader and comprehensive study of goal recognition methods in the literature.

## Data Availability Statement

The original contributions presented in the study are publicly available. This data can be found here: https://doi.org/10.5061/dryad.zkh1893b5. Code used in evaluation will also be made available at https://gitlab.com/pkthunder/frontiers-goal-rec-article.

## Author Contributions

IR, PK, JW, MR, and LH contributed to conception and design of the study and manuscript revision. IR and JW designed and implemented RAGeR. PK generated data and ran Elexir-MCTS and PANDA-Rec. IR performed statistical analyses. IR, PK, JW, and LH wrote sections of the manuscript. All authors read and approved the submitted version of the manuscript.

## Funding

MR and LH thank ONR and NRL for funding portions of this research. This material is based upon work supported by ONR and DARPA under Contract No. HR001119C0128. Any opinions, findings and conclusions or recommendations expressed in this material are those of the author(s) and do not necessarily reflect the views of DARPA, the Department of the Navy, Department of Defense, or the U.S. Government.

## Conflict of Interest

The authors declare that the research was conducted in the absence of any commercial or financial relationships that could be construed as a potential conflict of interest.

## Publisher's Note

All claims expressed in this article are solely those of the authors and do not necessarily represent those of their affiliated organizations, or those of the publisher, the editors and the reviewers. Any product that may be evaluated in this article, or claim that may be made by its manufacturer, is not guaranteed or endorsed by the publisher.

## Footnotes

^1^ https://www.uni-ulm.de/en/in/ki/research/software/panda

^2^ https://www.cs.rochester.edu/research/cisd/resources/monroe-plan/

^3^For reproducibility of our experiments, Elexir-MCTS was configured with an ϵ-greedy tree policy (ϵ = 0.5), a random playout policy, and a maximum number of search iterations of 200, 000 (high inspectability traces) and 30, 000 (medium and low inspectability traces). For RAGeR, the maximum search iterations was set to 10 for all traces in Minecraft, and 15 in Monroe. All experiments were run on standard desktop machines/servers; none of the presented methods required GPU resources.

^4^Hand edits were minor and only affected representation, not knowledge. For example, analogy requires that predicates have consistent arity and argument order, whereas PANDA-Rec does not.

^5^The no noise condition was left out of analyses due to a lack of variance.

^6^HTN can be found at https://github.com/panda-planner-dev/domains/blob/master/partial-order/Monroe/domain.hddl
